# Breast Cancer Susceptibility Gene Sequence Variations and Development of Contralateral Breast Cancer

**DOI:** 10.1001/jamanetworkopen.2024.52158

**Published:** 2024-12-30

**Authors:** Anne S. Reiner, Gordon P. Watt, Kathleen E. Malone, Charles F. Lynch, Esther M. John, Julia A. Knight, Meghan Woods, Xiaolin Liang, Marc Tischkowitz, David V. Conti, Mark E. Robson, Lene Mellemkjær, Sharon N. Teraoka, Patrick Concannon, Jonine L. Bernstein

**Affiliations:** 1 Department of Epidemiology and Biostatistics, Memorial Sloan Kettering Cancer Center, New York, New York; 2 Fred Hutchinson Cancer Research Center, Seattle, Washington; 3 University of Iowa, Iowa City; 4 Department of Epidemiology and Population Health, Stanford University School of Medicine, Stanford, California; 5 Department of Medicine (Oncology), Stanford University School of Medicine, Stanford, California; 6 Stanford Cancer Institute, Stanford University School of Medicine, Stanford, California; 7 Lunenfeld-Tanenbaum Research Institute, Sinai Health, Toronto, Ontario, Canada; 8 Dalla Lana School of Public Health, University of Toronto, Toronto, Ontario, Canada; 9 Department of Medical Genetics, National Institute for Health Research Cambridge Biomedical Research Centre, University of Cambridge, Cambridge, United Kingdom; 10 Department of Population and Public Health Sciences, Norris Comprehensive Cancer Center, University of Southern California, Los Angeles; 11 Department of Medicine, Memorial Sloan Kettering Cancer Center, New York, New York; 12 Danish Cancer Institute, Copenhagen, Denmark; 13 Genetics Institute and Department of Pathology, Immunology and Laboratory Medicine, University of Florida, Gainesville

## Abstract

**Importance:**

Heterogeneity in development of estrogen receptor (ER)-specific first primary breast cancer exists due to deleterious germline variants in moderate- to high-penetrance breast cancer susceptibility genes, but it is unknown if these associations occur in ER-specific CBC.

**Objective:**

To determine the association of deleterious germline variants in breast cancer susceptibility genes with ER-specific CBC development and whether ER status of the first primary breast cancer modifies these associations.

**Design, Setting, and Participants:**

This case-control study included CBC cases and matched unilateral breast cancer controls from The Women’s Environment, Cancer, and Radiation Epidemiology (WECARE) Study, a population-based case-control study. Eligible women were diagnosed between 1985 and 2000 with data and biospecimens collected from 2001 to 2004. Eligible participants were women younger than 55 years at first invasive breast cancer diagnosis. Participants were matched on age, diagnosis year, cancer registry region, and race and ethnicity, and countermatched on radiation treatment. For cases, CBC occurred 1 year or more following first breast cancer diagnosis. Analyses were performed from May to October 2024.

**Exposures:**

*CHEK2* 1100delC and deleterious variants in *ATM*, *BRCA1,* and *BRCA2*.

**Main Outcome and Measure:**

Development of CBC, measured as a rate ratio (RR).

**Results:**

A total of 1290 women were included in analysis (median [IQR] age at first diagnosis, 47 [42-51] years). The ER-positive CBC rate for women with deleterious *ATM* variants was 4 times higher than for women without deleterious *ATM* variants (RR, 4.84; 95% CI, 1.11-21.08; *P* = .04); no women with ER-negative CBC carried deleterious *ATM* variants. The ER-positive CBC rates for women with deleterious variants in *BRCA2* or *CHEK2* 1100delC were 5 to 6 times higher than for women without deleterious variants in *BRCA2* or *CHEK2* 1100delC, respectively (*BRCA2*: RR, 5.88; 95% CI, 2.61-13.26, *P* < .001; *CHEK2* 1100delC: RR, 6.06; 95% CI, 1.26-29.04; *P* = .02). The ER-negative CBC rate for women with deleterious *BRCA1* variants was 26 times higher than for women without deleterious *BRCA1* variants (RR, 26.16; 95% CI, 8.01-85.44; *P* < .001). First primary breast cancer ER status did not modify associations between deleterious variants and ER-specific CBC development.

**Conclusions and Relevance:**

In this case-control study of CBC, deleterious variants in breast cancer susceptibility genes were differentially associated with ER-specific CBC development. Germline variation profile may inform estimates of outcomes for ER-specific CBC subtypes.

## Key Points

**Question:**

Are moderate- to high-risk breast cancer susceptibility variants associated with development of estrogen receptor (ER)-positive and ER-negative contralateral breast cancer (CBC) subtypes?

**Findings:**

In this case-control study of 1290 women, the rate of ER-positive CBC was 5 to 6 times higher in women with deleterious variants in *BRCA2, ATM,* and *CHEK2*. The rate of ER-negative CBC was 26 times higher in women with deleterious *BRCA1* variants.

**Meaning:**

In this study, deleterious variants in breast cancer susceptibility genes were associated with differential rates of ER-specific CBC subtypes, providing further evidence of distinct etiologies of CBC subtypes.

## Teaser

This case-control study of women with breast cancer examines the risk of developing contralateral breast cancer subtypes associated with germline variants of breast cancer susceptibility genes.

## Introduction

Deleterious variants in *BRCA1, BRCA2,* and *ATM,* and the *CHEK2* 1100delC variant are associated with development of first primary breast cancer.^1,2,3,4^ We, and others, have shown similar associations with development of contralateral breast cancer (CBC).^5,6,7,8^ Recently, the Breast Cancer Association Consortium (BCAC) demonstrated different risk profiles for protein-truncating variants in breast cancer susceptibility genes according to breast cancer subtypes.^9^ The extent to which deleterious variants in breast cancer susceptibility genes are associated with development of CBC defined by estrogen receptor (ER) status is unknown, but clinically important. In this study, we examined whether deleterious variants in *ATM*, *BRCA1,* and *BRCA2*, and *CHEK2* 1100delC are associated with development of ER-positive CBC and ER-negative CBC. We also investigated whether ER status of the first primary breast cancer modifies these associations.

## Methods

### The WECARE Study

The Women’s Environment, Cancer, and Radiation Epidemiology (WECARE) Study is a 2-phase, population-based study of CBC cases and matched controls with unilateral breast cancer (UBC). This report focuses on phase I of the WECARE study, which included 708 CBC cases and 1399 matched UBC controls. Eligible women were identified by 5 population-based cancer registries in Denmark and the US (Iowa; Western region of Washington; Los Angeles County, California; and Orange and San Diego counties, California). The study protocol was approved by institutional review boards at each site and the ethics committee system in Denmark, and all participants provided written informed consent. The reporting of this study followed the Strengthening the Reporting of Observational Studies in Epidemiology (STROBE) reporting guideline for case-control studies.

Inclusion criteria,^10,11,12^ matching,^5,10,11^ and variant screening of *BRCA1/2*,^5^ *ATM*,^12^ and *CHEK2* 1100delC^6^ have been previously described in detail. In brief, all participants were diagnosed with their first primary breast cancer between January 1985 and December 1999 at age 54 years or younger. Eligible CBC cases had at least 1 year between their 2 breast cancer diagnoses, resided in the same reporting area for both diagnoses, and had not been diagnosed with any prior or intervening cancers (except for nonmelanoma skin cancer or in situ cervical cancer). Eligible UBC controls had no subsequent cancer diagnoses and no prophylactic mastectomy of the contralateral breast during the at-risk period. The at-risk period for each control was the same length as the interval between the first and second cancer diagnoses of her matched case. This at-risk period began on the control’s date of diagnosis and ended on the reference date defined by the end of her at-risk period.

Two controls were individually matched to each case on year of birth, year of diagnosis, cancer registry, and race and ethnicity. Self-reported race and ethnicity were assessed in the study for matching purposes and classified according to categories defined by the investigator. Race and ethnicity were considered for matching because (1) of the varying racial and ethnic distributions in the different regions where participants were recruited, and (2) prior work has shown associations of race and ethnicity with CBC rates. Racial and ethnic categories used for matching included Black, Chinese, Filipino, Japanese, other Asian, White (Hispanic and non-Hispanic), and other. Totals are not available to report due to confidentiality issues and compliance with EU country data protection regulations.

To improve statistical efficiency for the original study aim, case-control triplets were countermatched on cancer registry-reported radiation treatment.^1^ All eligible participants were alive at contact, able to provide written informed consent, provide a blood sample, and complete an interview, which included a structured questionnaire on variables known or suspected to be associated with development of breast cancer. Detailed methods for the laboratory analyses and quality control used to generate the variant data have been previously published.^5,6,12^ Briefly, from the collected blood samples, DNA was prepared by phenol-chloroform extraction. All coding exons of the *ATM* gene along with flanking intronic sequences and coding and flanking intronic regions of *BRCA1* and *BRCA2* were screened for variation by denaturing high-performance liquid chromatography followed by sequence confirmation.^5,12^ For detection of *CHEK2* 1100delC, an assay was developed based on ligation-dependent amplification. The reliability of the ligation assay was confirmed using denaturing gradient gel electrophoresis.^6^ Deleterious variants were defined as those estimated to result in loss of function, whether protein-truncating or missense (eTable 1 in Supplement 1).

Due to confidentiality issues and compliance with EU country data protection regulations, number of individuals fewer than 5 are not reported, and some numbers of individuals more than 4 are masked to prevent back-calculation of a number of individuals fewer than 5.

### Statistical Analyses

For participants with CBC, ER status of the second primary breast cancer defined the subtype-specific disease outcome; this was obtained from cancer registry records or by abstracting medical records. The unit of analysis was each set of the CBC case and the individually matched UBC controls. Multivariable rate ratios (RR) and 95% CIs were estimated as previously described^5,6,7^ by fitting conditional logistic regression models adjusted for variables known or suspected to be associated with development of CBC including age at first primary breast cancer, age at menarche, age at menopause 2 years prior to first primary breast cancer (to exclude treatment-induced menopause), number of full-term pregnancies as of first primary breast cancer diagnosis, first primary breast cancer therapies (chemotherapy, hormonal therapy, radiation treatment), and characteristics of the first primary breast cancer, including histology, stage, ER status, and progesterone receptor status. In addition, for *ATM* and *CHEK2* 1100delC models, known *BRCA1/2* deleterious variant status was also included. Likewise, for *BRCA1* models, *BRCA2* deleterious variant status was included, and for *BRCA2* models, *BRCA1* deleterious variant status was included. Models included a log-weight offset term to account for the countermatched design.^11^ To include all women, missing information on a covariable was represented by an indicator variable.^13^ Additionally, we formally tested the statistical interaction between ER status of the first breast cancer and deleterious variant status for each gene using models parameterized to preserve matched sets. For example, to estimate ER-positive CBC-specific RRs for *CHEK2* 1100delC separately for women with ER-positive first breast cancer and ER-negative first breast cancer, the model included 2 indicator variables for *CHEK2* 1100delC, one for ER-positive first breast cancer and one for ER-negative first breast cancer, as well as a variable for ER status of the first breast cancer and adjustment for other potential confounders. In a post hoc analysis, we further adjusted for first-degree family history of breast cancer (participants reporting having a mother, sister, or daughter with breast cancer) to investigate if estimates were sensitive to this additional covariable adjustment. All statistical tests were 2-sided with an α level of statistical significance .05. Analyses were performed in SAS version 9.4 (SAS Institute).

## Results

A total of 1290 participants were included for analyses (median [IQR] age at first breast cancer diagnosis, 47 [42-51] years), 434 cases with known CBC ER status and 856 matched controls (eTable 2 in Supplement 1). The median length of the at-risk period was 4.9 years (range, 1.0-15.6 years). There were 434 cases with known CBC ER status and 856 matched controls of whom 295 were women with ER-positive CBC and 580 were matched controls, and 139 were women with ER-negative CBC and 276 were matched controls (Table). Of 295 cases with ER-positive CBC, 41 women (14%) carried at least 1 deleterious *ATM*, *BRCA1*, or *BRCA2* variant, or *CHEK2* 1100delC, with approximately half of the 41 women carrying a deleterious variant in either *BRCA1* or *BRCA2*. Of 580 matched controls to ER-positive CBC cases, only 35 women (6%) carried at least 1 deleterious *ATM*, *BRCA1*, or *BRCA2* variant or *CHEK2* 1100delC. Correspondingly, of 139 cases with ER-negative CBC, 52 women (37%) carried at least 1 deleterious *BRCA1* or *BRCA2* variant, or *CHEK2* 1100delC, with the majority carrying a deleterious variant in either *BRCA1* or *BRCA2*. Of 276 matched controls, only 20 women (7%) carried at least 1 deleterious *ATM*, *BRCA1*, or *BRCA2* variant or *CHEK2* 1100delC.

**Table.** Variant Status and Association With ER-Specific CBC Risk in the WECARE Study

Variant type and carriership	ER-positive CBC				ER-negative CBC			
	Cases, No. (%) (n = 295)^a^^,^^b^	Matched UBC controls, No. (%) (n = 580)^a^^,^^b^	RR (95% CI)^c^	*P* value^c^	Cases, No. (%) (n = 139)^b^^,^^d^	Matched UBC controls, No. (%) (n = 276)^b^^,^^d^	RR (95% CI)^c^	*P* value^c^
*ATM*								
Deleterious noncarrier	287 (97.3)	<580	1 [Reference]	NA	139 (100.0)	<276	1 [Reference]	NA
Deleterious carrier	8 (2.7)	<5	4.84 (1.11-21.08)	.04	0	<5	NR^e^	NR
Protein-truncating carrier	<8	<5	4.27 (0.94-19.37)	.06	0	<5	NR^e^	NR
Deleterious missense carrier	<5	0	NR^e^	NR	0	0	NR^e^	NR
*BRCA1*								
Deleterious noncarrier	287 (97.3)	563 (97.1)	1 [Reference]	NA	93 (66.9)	268 (97.1)	1 [Reference]	NA
Deleterious carrier	<8	<17	0.61 (0.16-2.33)	.47	<47	8 (2.9)	26.16 (8.01-85.44)	<.001
Protein-truncating carrier	<7	<16	0.45 (0.10-2.03)	.30	<47	8 (2.9)	22.44 (6.68-75.34)	<.001
Deleterious missense carrier	<5	<5	2.04 (0.13-32.37)	.61	<47	0	NR^e^	NR
*BRCA2*								
Deleterious noncarrier	272 (92.2)	566 (97.6)	1 [Reference]	NA	<139	268 (97.1)	1 [Reference]	NA
Deleterious carrier	<23	<14	5.88 (2.61-13.26)	<.001	<7	8 (2.9)	1.83 (0.42-7.89)	.42
Protein-truncating carrier	<22	<13	7.73 (3.16-18.91	<.001	<7	<8	2.56 (0.53-12.42)	.24
Deleterious missense carrier	<5	<5	0.77 (0.06-10.39)	.85	0	<5	NR^e^	NR
*CHEK2*								
Noncarrier of 1100delC	290 (98.3)	<580	1 [Reference]	NA	<139	<276	1 [Reference]	NA
Carrier of 1100delC	5 (1.7)	<5	6.06 (1.26-29.04)	.02	<5	<5	0.67 (0.01-40.17)	.85

Abbreviations: CBC, contralateral breast cancer; ER, estrogen receptor; NA, not applicable; NR, not reported; RR, rate ratio; UBC, unilateral breast cancer; WECARE, Women’s Environment, Cancer, and Radiation Epidemiology.

^a^ ER-positive CBC cases with unknown variant status (<5 participants) and matched UBC controls with unknown variant status (<5 participants) are not listed as gene-specific categories but are included in total column counts.

^b^ Due to confidentiality issues and compliance with EU country data protection regulations, results fewer than 5 are not reported. Counts are indicated to be fewer than the reported total circumvent back calculation of reportable patients either in that category or another category, including gene-specific unknown variant status.

^c^ Models include age at first primary breast cancer diagnosis, chemotherapy and/or hormonal therapy, radiation treatment, characteristics of the first primary breast cancer (histology, stage, ER status, PR status), age at menarche, age at menopause 2 years prior to first primary breast cancer diagnosis, and number of full-term pregnancies as of the time of first primary breast cancer diagnosis. *ATM* and *CHEK2* 1100delC results are also adjusted for *BRCA1/2* deleterious variant carriership. *BRCA1* results are also adjusted for *BRCA2* deleterious variant carriership. *BRCA2* results are also adjusted for *BRCA1* deleterious variant carriership.

^d^ ER-negative CBC cases with unknown variant status (<5 participants) and matched UBC controls with unknown variant status (<5 participants) are not listed as gene-specific categories but are included in total column counts.

^e^ Could not be modeled due to a zero count of CBC cases, UBC controls, or both.

Carriers of an *ATM* deleterious variant had a 4 times higher rate of ER-positive CBC compared with the rate in noncarriers of an *ATM* deleterious variant (RR, 4.84; 95% CI, 1.11-21.08), which was driven primarily by protein-truncating *ATM* variants (RR, 4.27; 95% CI, 0.94-19.37) (Table). No women with ER-negative CBC had *ATM* deleterious variants.

Compared with the rate of ER-positive CBC in women without deleterious *BRCA2* variants, the rate was almost 6 times higher in women with deleterious *BRCA2* variants (RR, 5.88; 95% CI, 2.61-13.26), but the rate of ER-negative CBC was not significantly higher; conversely, compared with the rate of ER-negative CBC in women without deleterious *BRCA1* variants, the rate was 26 times higher in women with deleterious *BRCA1* variants (RR, 26.16; 95% CI, 8.01-85.44), but the rate of ER-positive CBC was not higher (Table). These RR estimates were driven by protein-truncating variants. Of note, of the 139 women with ER-negative CBC, which included carriers and noncarriers of deleterious variants, approximately one-third were *BRCA1* deleterious variant carriers. Furthermore, more than 75% of women with a *BRCA1* deleterious missense variant had ER-negative CBC. Likewise, the majority of women with a *BRCA1* protein-truncating variant had ER-negative CBC.

Compared with women without the *CHEK2* 1100delC variant, women carrying the *CHEK2* 1100delC variant had a 6 times higher rate of ER-positive CBC (RR, 6.06; 95% CI, 1.26-29.04), but the rate of ER-negative CBC was not higher, although CIs were wide. Finally, associations of gene-specific deleterious variants with development of ER-positive CBC and ER-negative CBC were not statistically significantly modified by first primary ER status (results not shown). In a post hoc analysis that additionally adjusted for first-degree family history of breast cancer, model estimates did not appreciably change; magnitude, direction, and statistical significance of all model estimates were unchanged.

## Discussion

With the growing availability of next-generation sequencing and multigene panel testing, health care professionals and women diagnosed with breast cancer increasingly seek to incorporate this information into shared clinical decision-making.^14,15,16^ We examined development of ER-positive and ER-negative tumor subtype–specific CBC for women carrying deleterious variants in moderate to high-risk breast cancer susceptibility genes. Development of ER-specific CBC differed depending on the breast cancer susceptibility gene.

Our results extend those from BCAC and the Consortium of Investigators of Modifiers of *BRCA1/2* (CIMBA) investigations of first primary breast cancers. Results from CIMBA demonstrated that ER-positive primary cancers were more likely to occur in patients with mutations in *BRCA2* than *BRCA1*.^17^ We similarly demonstrate this increased risk in the current study in the setting of CBC. Results from BCAC demonstrated that *CHEK2* variants were associated with all breast cancer subtypes except triple negative.^18^ Analogously, we demonstrated an association between carrying *CHEK2* 1100delC and development of ER-positive CBC, but not ER-negative CBC. BCAC demonstrated that carriers of protein-truncating variants in *BRCA1* had a higher rate of ER-negative first primary breast cancer and that carriers of protein-truncating variants in *ATM* and *CHEK2* had higher rates of ER-positive first primary breast cancer.^9^ However, whereas BCAC reported a statistically significant association with *BRCA1* protein-truncating variants for development of both ER-positive and ER-negative first primary breast cancer, we only observed a statistically significant association with *BRCA1* protein-truncating variants for development of ER-negative CBC in the current study. Similarly, for carriers of *BRCA2* protein-truncating variants, we only observed a statistically significant association with *BRCA2* protein-truncating variants for development of ER-positive CBC. It is possible that the current analysis was underpowered, or that the differences in study results are due to differing designs. The WECARE Study compares women with a first primary breast cancer and a CBC and women with only a first primary breast cancer, whereas the BCAC study compared women with a first primary breast cancer and women with no breast cancer. However, the study of second primary cancers has been shown to be an efficient method for evaluating the associations with rare genetic susceptibility markers.^19^ A strength of the current study is that variant screening was conducted after breast cancer diagnosis, and therefore, treatment decisions that could impact CBC risk were independent of carrier status.

We and others have found high concordance between hormone receptor (HR) status of first and second primary breast cancers^20,21,22^ that may reflect an individual’s exposure to hormonal or other exogenous or endogenous risk factors. Considering the results herein, along with previously reported parallel associations with first primary breast cancer subtypes, concordance may reflect an underlying genetic susceptibility.

### Limitations

This study has several limitations. Although our population-based study was large (in the CBC context) with long at-risk periods, the relatively small number of carriers with deleterious variants and CBC cases with known ER status resulted in wide CIs. Genotyping was not performed for other *CHEK2* variants besides 1100delC and there were too few carriers of *PALB2* deleterious variants, a gene for which deleterious variants are known to be associated with development of CBC, to report.^9^ Additionally, HR status was determined in pathology departments of treating hospitals, not centrally using a standardized protocol. However, prior work demonstrates good agreement between cancer registry-reported HR status and HR status determined by a single academic reference laboratory.^23^ Finally, deleterious missense variants are rare relative to protein-truncating variants in *ATM* and *BRCA1* or *BRCA2*. As a result, statistical power to assess their relationship to CBC HR status was limited.

## Conclusions

We found that breast cancer susceptibility variants in *BRCA1, BRCA2*, *ATM*, and *CHEK2* were associated with development of CBC in a subtype-specific manner. Germline variation profile, determined at first primary breast cancer diagnosis, can improve individualized estimates of development of CBC to inform screening and decisions pertaining to preventive surgeries and other treatment options.

## Notes

Supplement 1. **eTable 1.** Variant Descriptions and Classifications **eTable 2.** The WECARE Study Characteristics by Case-Control Status

Supplement 2.
Data Sharing Statement

## References

[1] Johnson N, Fletcher O, Palles C, . Counting potentially functional variants in BRCA1, BRCA2 and ATM predicts breast cancer susceptibility. Hum Mol Genet. 2007;16(9):1051-1057. doi:10.1093/hmg/ddm050 17341484

[2] CHEK2 Breast Cancer Case-Control Consortium. CHEK2*1100delC and susceptibility to breast cancer: a collaborative analysis involving 10,860 breast cancer cases and 9,065 controls from 10 studies. Am J Hum Genet. 2004;74(6):1175-1182. doi:10.1086/421251 15122511 PMC1182081

[3] Fletcher O, Johnson N, dos Santos Silva I, ; kConFab Investigators; AOCS Group; GENICA Consortium; Breast Cancer Association Consortium. Missense variants in ATM in 26,101 breast cancer cases and 29,842 controls. Cancer Epidemiol Biomarkers Prev. 2010;19(9):2143-2151. doi:10.1158/1055-9965.EPI-10-0374 20826828 PMC2938473

[4] Renwick A, Thompson D, Seal S, ; Breast Cancer Susceptibility Collaboration (UK). ATM mutations that cause ataxia-telangiectasia are breast cancer susceptibility alleles. Nat Genet. 2006;38(8):873-875. doi:10.1038/ng1837 16832357

[5] Malone KE, Begg CB, Haile RW, . Population-based study of the risk of second primary contralateral breast cancer associated with carrying a mutation in BRCA1 or BRCA2. J Clin Oncol. 2010;28(14):2404-2410. doi:10.1200/JCO.2009.24.2495 20368571 PMC2881721

[6] Mellemkjaer L, Dahl C, Olsen JH, ; WECARE Study Collaborative Group. Risk for contralateral breast cancer among carriers of the CHEK2*1100delC mutation in the WECARE Study. Br J Cancer. 2008;98(4):728-733. doi:10.1038/sj.bjc.6604228 18253122 PMC2259175

[7] Reiner AS, Robson ME, Mellemkjær L, ; WECARE Study Collaborative Group. Radiation treatment, ATM, BRCA1/2, and CHEK2*1100delC pathogenic variants and risk of contralateral breast cancer. J Natl Cancer Inst. 2020;112(12):1275-1279. doi:10.1093/jnci/djaa031 32119081 PMC7735763

[8] Molina-Montes E, Pérez-Nevot B, Pollán M, Sánchez-Cantalejo E, Espín J, Sánchez MJ. Cumulative risk of second primary contralateral breast cancer in BRCA1/BRCA2 mutation carriers with a first breast cancer: a systematic review and meta-analysis. Breast. 2014;23(6):721-742. doi:10.1016/j.breast.2014.10.005 25467311

[9] Dorling L, Carvalho S, Allen J, Gonzalez-Neira A, Luccarini C, ; Breast Cancer Association Consortium. Breast cancer risk genes—association analysis in more than 113,000 women. N Engl J Med. 2021;384:428-439. doi:10.1056/NEJMoa1913948 33471991 PMC7611105

[10] Stovall M, Smith SA, Langholz BM, ; Women’s Environmental, Cancer, and Radiation Epidemiology Study Collaborative Group. Dose to the contralateral breast from radiotherapy and risk of second primary breast cancer in the WECARE study. Int J Radiat Oncol Biol Phys. 2008;72(4):1021-1030. doi:10.1016/j.ijrobp.2008.02.040 18556141 PMC3782859

[11] Bernstein JL, Langholz B, Haile RW, ; WECARE study. Study design: evaluating gene-environment interactions in the etiology of breast cancer—the WECARE study. Breast Cancer Res. 2004;6(3):R199-R214. doi:10.1186/bcr771 15084244 PMC400669

[12] Bernstein JL, Haile RW, Stovall M, ; WECARE Study Collaborative Group. Radiation exposure, the ATM Gene, and contralateral breast cancer in the women’s environmental cancer and radiation epidemiology study. J Natl Cancer Inst. 2010;102(7):475-483. doi:10.1093/jnci/djq055 20305132 PMC2902825

[13] Huberman M, Langholz B. Application of the missing-indicator method in matched case-control studies with incomplete data. Am J Epidemiol. 1999;150(12):1340-1345. doi:10.1093/oxfordjournals.aje.a009966 10604777

[14] Boddicker NJ, Hu C, Weitzel JN, . Risk of late-onset breast cancer in genetically predisposed women. J Clin Oncol. 2021;39(31):3430-3440. doi:10.1200/JCO.21.00531 34292776 PMC8547938

[15] Yadav S, Hu C, Nathanson KL, . Germline pathogenic variants in cancer predisposition genes among women with invasive lobular carcinoma of the breast. J Clin Oncol. 2021;39(35):3918-3926. doi:10.1200/JCO.21.00640 34672684 PMC8660003

[16] Tung N, Desai N. Germline genetic testing for women with breast cancer: shifting the paradigm from whom to test to whom NOT to test. J Clin Oncol. 2021;39(31):3415-3418. doi:10.1200/JCO.21.01761 34491781

[17] Mavaddat N, Barrowdale D, Andrulis IL, ; HEBON; EMBRACE; GEMO Study Collaborators; kConFab Investigators; SWE-BRCA Collaborators; Consortium of Investigators of Modifiers of BRCA1/2. Pathology of breast and ovarian cancers among BRCA1 and BRCA2 mutation carriers: results from the Consortium of Investigators of Modifiers of BRCA1/2 (CIMBA). Cancer Epidemiol Biomarkers Prev. 2012;21(1):134-147. doi:10.1158/1055-9965.EPI-11-0775 22144499 PMC3272407

[18] Mavaddat N, Dorling L, Carvalho S, Allen J, Gonzalez-Neira A, ; Breast Cancer Association Consortium. Pathology of tumors associated with pathogenic germline variants in 9 breast cancer susceptibility genes. JAMA Oncol. 2022;8(3):e216744. https://pubmed.ncbi.nlm.nih.gov/35084436/35084436 10.1001/jamaoncol.2021.6744 PMC8796069

[19] Begg CB, Berwick M. A note on the estimation of relative risks of rare genetic susceptibility markers. Cancer Epidemiol Biomarkers Prev. 1997;6(2):99-103. 9037560

[20] Swain SM, Wilson JW, Mamounas EP, . Estrogen receptor status of primary breast cancer is predictive of estrogen receptor status of contralateral breast cancer. J Natl Cancer Inst. 2004;96(7):516-523. doi:10.1093/jnci/djh097 15069113

[21] Huo D, Melkonian S, Rathouz PJ, Khramtsov A, Olopade OI. Concordance in histological and biological parameters between first and second primary breast cancers. Cancer. 2011;117(5):907-915. doi:10.1002/cncr.25587 20945326 PMC3022996

[22] Reiner AS, Lynch CF, Sisti JS, ; WECARE Study Collaborative Group. Hormone receptor status of a first primary breast cancer predicts contralateral breast cancer risk in the WECARE study population. Breast Cancer Res. 2017;19(1):83. doi:10.1186/s13058-017-0874-x 28724391 PMC5517810

[23] Ma H, Wang Y, Sullivan-Halley J, . Breast cancer receptor status: do results from a centralized pathology laboratory agree with SEER registry reports? Cancer Epidemiol Biomarkers Prev. 2009;18(8):2214-2220. doi:10.1158/1055-9965.EPI-09-0301 19661080 PMC3782852

