# Supplementary material for: Breast Cancer Susceptibility Gene Sequence Variations and Development of Contralateral Breast Cancer
Source: JAMA Netw Open. 2024 Dec 30;7(12):e2452158. doi: 10.1001/jamanetworkopen.2024.52158 (PMC11686411; doi:10.1001/jamanetworkopen.2024.52158)
Supplement: Supplement 1. — eTable 1. Variant Descriptions and Classifications eTable 2. The WECARE Study Characteristics by Case-Control Status [file jamanetwopen-e2452158-s001.pdf]

Supplemental Online Content

Reiner AS, Watt GP, Malone KE, et al. Breast cancer susceptibility gene sequence variations and development of contralateral breast cancer. *JAMA Netw Open.* 2024;7(12):e2452158. doi:10.1001/jamanetworkopen.2024.52158

eTable 1. Variant Descriptions and Classifications

eTable 2. The WECARE Study Characteristics by Case-Control Status

This supplemental material has been provided by the authors to give readers additional information about their work.

© 2024 Reiner AS et al. *JAMA Network Open.*

eTable 1. Variant Descriptions and Classifications

Gene	HGVS Variant Description[a]			Variant Classification[b]	
	Nucleotide	Protein	dpSNP	Protein-truncating	Deleterious Missense
ATM	NM_000051.3:c.742C>T	NP_000042.3:p.Arg248*	rs730881336	X	
ATM	NM_000051.3:c.1607+1G>T		rs772926890	X	
ATM	NM_000051.4:c.5177+2T>G			X	
ATM	NM_000051.3:c.5405dup	NP_000042.3:p.His1802Glnfs*2	rs1555106452	X	
ATM	NM_000051.3:c.5712dup	NP_000042.3:p.Ser1905Ilefs*25	rs587781730	X	
ATM	NM_000051.3:c.6200C>A	NP_000042.3:p.Ala2067Asp	rs397514577		X
ATM	NM_000051.3:c.6908dup	NP_000042.3:p.Glu2304Glyfs*69	rs773570504	X	
BRCA1	NM_007294.4:c.68_69delAG	NP_009225.1:p.Glu23Valfs*17	rs80357914	X	
BRCA1	NM_007294.4:c.68_69dup	NP_009225.1:p.Cys24Serfs*8	rs80357914	X	
BRCA1	NM_007294.4:c.83_84del	NP_009225.1:p.Leu28Argfs*12	rs80357728	X	
BRCA1	NM_007294.4:c.178_179del	NP_009225.1:p.Gln60Valfs*5	rs397508907	X	
BRCA1	NM_007294.4:c.181T>G	NP_009225.1:p.Cys61Gly	rs28897672		X
BRCA1	NM_007294.4:c.211A>G	NP_009225.1:p.Arg71Gly	rs80357382		X
BRCA1	NM_007294.3:c.302-2del		rs273899695	X	
BRCA1	NM_007294.4:c.594-2A>C		rs80358033	X	
BRCA1	NM_007294.4:c.1121del	NP_009225.1:p.Thr374Asnfs*2	rs80357612	X	
BRCA1	NM_007294.4:c.1529C>A	NP_009225.1:p.Ser510*	rs80357427	X	
BRCA1	NM_007294.4:c.1556del	NP_009225.1:p.Lys519Argfs*13	rs80357662	X	
BRCA1	NM_007294.4:c.1687C>T	NP_009225.1:pGln563*	rs80356898	X	
BRCA1	NM_007294.4:c.2314del	NP_009225.1:p.Val772Tyrfs*20	rs80357957	X	
BRCA1	NM_007294.4:c.2475del	NP_009225.1:p.Asp825Glufs*21	rs80357970	X	
BRCA1	NM_007294.4:c.2515del	NP_009225.1:p.His839Thrfs*7	rs80357607	X	
BRCA1	NM_007294.4:c.2635G>T	NP_009225.1:p.Glu879*	rs80357251	X	
BRCA1	NM_007294.4:c.2679_2682del	NP_009225.1:p.Lys893Asnfs*106	rs80357596	X	
BRCA1	NM_007294.4:c.3029_3030del	NP_009225.1:p.Pro1010Argfs*7	rs80357510	X	
BRCA1	NM_007294.4:c.3481_3491del	NP_009225.1:p.Glu1161Phefs*3	rs80357877	X	

© 2024 Reiner AS et al. *JAMA Network Open.*

	HGVS Variant Description[a]			Variant Classification[b]	
Gene	Nucleotide	Protein	dpSNP	Protein-truncating	Deleterious Missense
BRCA1	NM_007294.4:c.3748G>T	NP_009225.1:p.Glu1250*	rs28897686	X	
BRCA1	NM_007294.4:c.3756_3759del	NP_009225.1:p.Ser1253Argfs*10	rs80357868	X	
BRCA1	NM_007294.4:c.4065_4068del	NP_009225.1:p.Asn1355Lysfs*10	rs80357508	X	
BRCA1	NM_007294.4:c.4183C>T	NP_009225.1:p.Gln1395*	rs80357260	X	
BRCA1	NM_007294.4:c.4251_4252del	NP_009225.1:p.Leu1418Argfs*9	rs80357977	X	
BRCA1	NM_007294.4:c.4327C>T	NP_009225.1:p.Arg1443*	rs41293455	X	
BRCA1	NM_007294.4:c.4357+1G>T		rs80358027	X	
BRCA1	NM_007297.4:c.4845+2T>C		rs397509210	X	
BRCA1	NM_007294.4:c.5123C>A	NP_009225.1:p.Ala1708Glu	rs28897696		X
BRCA1	NM_007294.4:c.5230del	NP_009225.1:p.Arg1744Glufs*21	rs397509240	X	
BRCA1	NM_007294.4:c.5266dup	NP_009225.1:p.Gln1756Profs*74	rs80357906	X	
BRCA1	NM_007294.4:c.5324T>G	NP_009225.1:p.Met1775Arg	rs41293463		X
BRCA1	NM_007294.4:c.5503C>T	NP_009225.1:p.Arg1835*	rs41293465	X	
BRCA1	NM_007294.4:c.5558dup	NP_009225.1:p.Tyr1853*	rs80357629	X	
BRCA2	NM_000059.4:c.51_52del	NP_000050.3:p.Arg18Leufs*12	rs80359483	X	
BRCA2	NM_000059.3:c.364del	NP_000050.2:p.Thr122Leufs*14s	rs397507680	X	
BRCA2	NM_000059.4:c.755_758del	NP_000050.3:p.Asp252Valfs*24	rs80359659	X	
BRCA2	NM_000059.4:c.1813dup	NP_000050.3:p.Ile605Asnfs*11	rs80359306	X	
BRCA2	NM_000059.3:c.2254_2257delGACT	NP_000050.3:p.Asp752Phefs*19	rs80359326	X	
BRCA2	NM_000059.4:c.2808_2811del	NP_000050.3:p.Ala938Profs*21	rs80359351	X	
BRCA2	NM_000059.4:c.3109C>T	NP_000050.3:p.Gln1037*	rs80358557	X	
BRCA2	NM_000059.3:c.3264dupT	NP_000050.2:p.Gln1089Serfs*10	rs80359380	X	
BRCA2	NM_000059.4:c.3847_3848del	NP_000050.3:p.Val1283Lysfs*2	rs80359405	X	
BRCA2	NM_000059.3:c.3865_3868delAAAT	NP_000050.2:p.Lys1289Alafs*3	rs80359412	X	
BRCA2	NM_000059.3:c.3978_3979insTTGC	NP_000050.2:p.Ala1327Leufs*4	rs1555283488	X	
BRCA2	NM_000059.3:c.4449delA	NP_000050.2:p.Asp1484Thrfs*2	rs80359448	X	
BRCA2	NM_000059.3:c.4936_4939delGAAA	NP_000050.2:p.Glu1646Glnfs*23	rs80359473	X	
BRCA2	NM_000059.4:c.5576_5579del	NP_000050.3:p.Ile1859Lysfs*3	rs80359520	X	

© 2024 Reiner AS et al. *JAMA Network Open.*

Gene	HGVS Variant Description[a]			Variant Classification[b]	
	Nucleotide	Protein	dpSNP	Protein-truncating	Deleterious Missense
BRCA2	NM_000059.4:c.5645C>A	NP_000050.3:p.Ser1882*	rs80358785	X	
BRCA2	NM_000059.4:c.5722_5723del	NP_000050.3:p.Leu1908Argfs*2	rs80359530	X	
BRCA2	NM_000059.4:c.5946del	NP_000050.3:p.Ser1982Argfs*22	rs80359550	X	
BRCA2	NM_000059.4:c.6275_6276del	NP_000050.3:p.Leu2092Profs*7	rs11571658	X	
BRCA2	NM_000059.4:c.7025_7026del	NP_000050.3:p.Gln2342Argfs*17	rs80359634	X	
BRCA2	NM_000059.3:c.7067_7068del	NP_000050.2:p.Phe2356Serfs*3	rs397507894	X	
BRCA2	NM_000059.3:c.7210_7216delinsTG	NP_000050.2:p.Lys2404Cysfs*6	rs397507904	X	
BRCA2	NM_000059.4:c.7558C>T	NP_000050.3:p.Arg2520*	rs80358981	X	
BRCA2	NM_000059.3:c.8322dupT	NP_000050.3:p.Met2775Tyrfs*7	rs80359706	X	
BRCA2	NM_000059.3:c.8970G>A	NP_000050.2:p.Trp2990*	rs80359149	X	
BRCA2	NM_000059.4:c.9097dup	NP_000050.3:p.Thr3033Asnfs*11	rs397507419	X	
BRCA2	NM_000059.4:c.9154C>T	NP_000050.3:p.Arg3052Trp	rs45580035		X
BRCA2	NM_000059.4:c.9253dup	NP_000050.3:p.Thr3085Asnfs*26	rs80359752	X	
BRCA2	NM_000059.4:c.9382C>T	NP_000050.3:p.Arg3128*	rs80359212	X	
BRCA1	NM_007294.4:c.4689C>G	NP_009225.1:p.Tyr1563*	rs80357433	X	
BRCA1	NM_007294.4:c.4484+1G>A		rs80358063	X	
BRCA1	NM_007294.4:c.213-12A>G		rs80358163	X	

[a]Variants from samples originating from Denmark are not listed due to privacy issues in compliance with data protection regulations.
[b]Protein-truncating variants and deleterious missense variants comprise the deleterious variants group.

© 2024 Reiner AS et al. *JAMA Network Open.*

eTable 2. Demographic, Tumor, and Treatment Characteristics by ER Specific Case and Matched Control Status

Variable	Category	ER+ CBC (n=295)	Matched UBC (n=580)	ER- CBC (n=139)	Matched UBC (n=276)
		**No. (%)[a]**	**No. (%)[a]**	**No. (%)[a]**	**No. (%)[a]**
Age at first primary breast cancer diagnosis	Continuous, years	48 (42-52)	47 (43-52)	45 (38-50)	45 (38-50)
Age at menarche	13+ years old (or never had period)	156 (52.9)	329 (56.7)	70 (50.4)	154 (55.8)
	<13 years old	139 (47.1)	251 (43.3)	69 (49.6)	122 (44.2)
Age at menopause	Premenopausal (or unknown)	216 (73.2)	419 (72.2)	103 (74.1)	207 (75.0)
	<45 years old	36 (12.2)	92 (15.9)	21 (15.1)	44 (15.9)
	45+ years old	43 (14.6)	69 (11.9)	15 (10.8)	25 (9.1)
Number of full-term pregnancies	None (or unknown)	55 (18.6)	94 (16.2)	24 (17.3)	48 (17.4)
	1	54 (18.3)	75 (12.9)	26 (18.7)	51 (18.5)
	2	109 (37.0)	223 (38.5)	46 (33.1)	103 (37.3)
	3	53 (18.0)	128 (22.1)	29 (20.9)	44 (15.9)
	4+	24 (8.1)	60 (10.3)	14 (10.1)	30 (10.9)
Stage of first primary breast cancer	Local	203 (68.8)	378 (65.2)	100 (71.9)	171 (62.0)
	Regional	92 (31.2)	202 (34.8)	39 (28.1)	105 (38.0)
Histology of first primary breast cancer	Lobular	52 (17.6)	56 (9.7)	6 (4.3)	25 (9.1)
	Other	243 (82.4)	524 (90.3)	133 (95.7)	251 (90.9)
Hormone therapy for the first primary breast cancer	No (or unknown)	202 (68.5)	382 (65.9)	111 (79.9)	179 (64.9)
	Yes	93 (31.5)	198 (34.1)	28 (20.1)	97 (35.1)
Chemotherapy for the first primary breast cancer	No	178 (60.3)	271 (46.7)	60 (43.2)	115 (41.7)
	Yes	117 (39.7)	309 (53.3)	79 (56.8)	161 (58.3)
Family history of breast cancer	No (or adopted)	204 (69.2)	466 (80.3)	95 (68.3)	228 (82.6)
	Yes	91 (30.9)	114 (19.7)	44 (31.7)	48 (17.4)

[a]For continuous variables, the median and interquartile range are presented as: median (interquartile range).

© 2024 Reiner AS et al. *JAMA Network Open.*
