# Supplementary material for: Breast Cancer Susceptibility Gene Sequence Variations and Development of Contralateral Breast Cancer
Source: JAMA Netw Open. 2024 Dec 30;7(12):e2452158. doi: 10.1001/jamanetworkopen.2024.52158 (PMC11686411; doi:10.1001/jamanetworkopen.2024.52158)
Supplement: Supplement 2. — Data Sharing Statement [file jamanetwopen-e2452158-s002.pdf]

Data Sharing Statement

Reiner. Breast Cancer Susceptibility Gene Sequence Variations and Development of Contralateral Breast Cancer. *JAMA Netw Open*. Published December 30, 2024. doi:10.1001/jamanetworkopen.2024.52158

Data
Data available: No

Additional Information
Explanation for why data not available: The data underlying this article cannot be shared publicly since the authors do not have permission to share the data.
